# Association between Intra- and Extra-Cellular Water Ratio Imbalance and Natriuretic Peptides in Patients Undergoing Hemodialysis

**DOI:** 10.3390/nu15051274

**Published:** 2023-03-03

**Authors:** Yui Nakayama, Yosuke Yamada, Shingo Ishii, Mai Hitaka, Keisuke Yamazaki, Motoyuki Masai, Nobuhiko Joki, Ken Sakai, Yasushi Ohashi

**Affiliations:** 1 Department of Nephrology, Toho University Graduate School of Medicine, Tokyo 143-8540, Japan; 2 Department of Nephrology, Toho University Omori Medical Center, Tokyo 143-8541, Japan; 3 Department of Physical Activity Research, National Institutes of Biomedical Innovation, Health and Nutrition, Osaka 566-0002, Japan; 4 Department of Nephrology, Toho University Sakura Medical Center, Sakura 285-0841, Japan; 5 Department of Urology, Mihama Hospital, Chiba 261-0013, Japan; 6 Department of Nephrology, Toho University Ohashi Medical Center, Tokyo 153-8515, Japan

**Keywords:** body composition, fluid volume overlord, dry weight, extracellular water, intracellular water, overhydration, natriuretic peptide, malnutrition, sarcopenia

## Abstract

Natriuretic peptides are associated with malnutrition and volume overload. Over-hydration cannot simply be explained by excess extracellular water in patients undergoing hemodialysis. We assessed the relationship between the extracellular and intracellular water (ECW/ICW) ratio, *N*-terminal pro-B-type natriuretic peptide (NT-proBNP), human atrial natriuretic peptide (hANP), and echocardiographic findings. Body composition was examined by segmental multi-frequency bioelectrical impedance analysis in 368 patients undergoing maintenance dialysis (261 men and 107 women; mean age, 65 ± 12 years). Patients with higher ECW/ICW ratio quartiles tended to be older, were on dialysis longer, and had higher post-dialysis blood pressure and lower body mass index, ultrafiltration volume, serum albumin, blood urea nitrogen, and creatinine levels (*p* < 0.05). The ECW/ICW ratio significantly increased with decreasing ICW, but not with ECW. Patients with a higher ECW/ICW ratio and lower percent fat had significantly higher natriuretic peptide levels. After adjusting for covariates, the ECW/ICW ratio remained an independent associated factor for natriuretic peptides (β = 0.34, *p* < 0.001 for NT-proBNP and β = 0.40, *p* < 0.001 for hANP) and the left ventricular mass index (β = 0.20, *p* = 0.002). The ICW-ECW volume imbalance regulated by decreased cell mass may explain the reserve capacity for fluid accumulation in patients undergoing hemodialysis.

## 1. Introduction

Chronic fluid volume overload occurs in 27–46% of patients undergoing hemodialysis [1,2,3] and is a major risk factor for cardiovascular events [4] and mortality [2,3]. Hypovolemia leads to intra- and post-dialysis hypotensive symptoms (muscle cramps, yawning, nausea, vomiting, dizziness, and syncope). Therefore, controlling the fluid volume within an optimal range is crucial for improving cardiovascular stress, quality of life, and survival. Optimal fluid volume status is usually described as dry weight in patients undergoing hemodialysis; it is the lowest tolerated post-dialysis weight achieved via gradual change, at which there are minimal signs or symptoms of hypovolemia or hypervolemia [5].

Total body water (TBW) is measured using the dilution method with deuterium (^2^H) or heavy oxygen (^18^O). TBW changes have been examined during short-term (≤10 days) weight loss or weight gain using the dilution method [6,7,8,9]. However, the dilution method is not practical for monitoring daily fluid volume changes in patients undergoing hemodialysis. Multi-frequency bioelectrical impedance analysis (MF-BIA) or bioelectrical impedance spectroscopy (BIS) are alternative methods for estimating fluid volume status, determining body composition, and monitoring changes over time in body composition [10,11]. MF-BIA- or BIS-guided fluid volume management reduces blood pressure (BP) and post-dialysis weight; however, this does not seem to improve patient survival [12]. Therefore, fluid volume overload cannot be simply explained as excess extracellular water (ECW) due to oral sodium and water intake, which presents as inter-dialysis weight gain (IDWG).

The balance between intracellular water (ICW) and ECW might change when the hydration component gradually decreases with aging or muscle attenuation, primarily due to decreased cell volume [13,14]. Sodium retention typically causes extracellular volume expansion and compensatory release of natriuretic peptides due to stretching of the cardiac wall. Elevated cardiac peptide levels might also be associated with malnutrition and fluid volume overload [15,16].

We hypothesized that individuals with fluid volume imbalance might have different reserve capacities for fluid accumulation. Recognizing these changes in body composition due to aging and sarcopenia can aid clinical decision-making for dry weight. Thus, we aimed to assess the relationship between the ECW/ICW ratio and natriuretic peptide levels to explore a novel marker for assessing the reserve capacity for fluid accumulation in patients undergoing hemodialysis.

## 2. Materials and Methods

### 2.1. Study Design and Participants

A multicenter, cross-sectional study was conducted in four maintenance hemodialysis clinics (Seijinkai Mihama Hospital, Seijinkai Mihama Sakura Clinic, Seijinkai Narita Clinic, and Seijinkai Katori Clinic) between 2019 and 2022. During this period, 1094 patients underwent hemodialysis in these centers (average age, 68 ± 13 years; 777 men and 317 women). Eligible participants were selected from patients undergoing daytime dialysis and identified from the electronic medical records. Adults (aged ≥ 20 years) on maintenance dialysis for ≥90 days who had a stable dialysis prescription for at least 30 days at the time of recruitment were eligible. Ultimately, 368 patients who provided informed consent were included in the present study. Patients were excluded if they met any of the following criteria: had undergone coronary and/or valvular intervention or had suffered a myocardial infarction within the last 6 months; had been hospitalized for unscheduled dialysis for the treatment of heart failure in the last 6 months; had echocardiographic evidence of a left ventricular ejection fraction (LVEF) < 40%; had a contraindication to bioimpedance measurement, such as a pacemaker, joint replacement, or mechanical heart valve; were pregnant; or had major amputations, advanced malignancy, or dementia. This study was approved by the Ethics Committee of Toho University Sakura Medical Center, Tokyo, Japan (Approval Number: S21073|Date: 26 April 2022 [S18086|27 December 2018]) and was conducted in accordance with the Declaration of Helsinki. Informed consent was obtained from all the participants.

### 2.2. Data Collection

The following baseline data were recorded: age, sex, anthropometric measurements, presence of diabetes mellitus (DM), hemodialysis vintage, body weight, and pre- and post-dialysis BP. The following standard laboratory parameters were collected during the long-interval hemodialysis session at the beginning of the month: serum albumin, blood urea nitrogen, serum creatinine, sodium, potassium, chloride, calcium, phosphorus, uric acid, glucose, C-reactive protein, hemoglobin, and intact parathyroid hormone (iPTH) levels. Dialysis adequacy, assessed in terms of the urea reduction ratio and single-pool Kt/Vurea, was measured using the Shinzato formula [17]. The geriatric nutritional risk index (GNRI) was calculated as (14.89 + albumin (g/dL)) + (41.7 × body weight/ideal body weight). The ideal body weight was calculated using height and an idealized body mass index (BMI) of 22 kg/m^2^ [18]. Pre-dialysis *N*-terminal pro-B-type natriuretic peptide (NT-proBNP) levels were determined using an electrochemiluminescence immunoassay system (Cobas8000 e801 module; Roche Diagnostics K.K., Tokyo, Japan). Post-dialysis human atrial natriuretic peptide (hANP) levels were determined using a chemiluminescent enzyme immunoassay system (CL-JACK RK; Minaris Medical Co., Ltd., Tokyo, Japan). Pre-dialysis chest X-ray was obtained to evaluate the cardio-thoracic index (CTR). CTR was calculated as the ratio of the maximum transverse cardiac diameter (mm) to the maximum thoracic diameter (mm). As a routine clinical practice, annual transthoracic echocardiographic examinations were performed by a single experienced cardiologist [date difference between body fluid composition analysis and echocardiographic examinations, median (10th–90th percentile) of −70 days (−275 to 68 days)]. The left atrial diameter (LAD), left ventricular end-diastolic diameter (LVDd), left ventricular end-systolic diameter (LVDs), left ventricular posterior wall thickness (PWT), interventricular septum thickness (IVST), and LVEF were measured. Left ventricular mass (LVM) was calculated using the Devereux Equation [19], and the LVM index (LVMI) was calculated as the ratio of LVM to body surface area (BSA).

### 2.3. Assessment of Body Fluid Composition

Standard MF-BIA was performed with the patient in the supine position on a flat non-conductive bed after hemodialysis. For body composition measurements, we used a segmental MF-BIA instrument (Inbody S10^®^; InBody Co., Ltd., Seoul, Korea; https://inbodyusa.com/ (accessed on 26 April 2022)) with eight tactile electrodes. The microprocessor-controlled switches and impedance analyzer were activated and the segmental resistances of the arms, trunk, and legs were measured at four frequencies (5, 50, 250, and 500 kHz). A total of 20 segment resistances were obtained for each individual. Subsequently, the sum of each body segment measurement was used to calculate the TBW, ICW, and ECW using MF-BIA software (Inbody S10^®^; InBody Co., Ltd., Seoul, Korea; https://inbodyusa.com/ (accessed on 26 April 2022)). Participants were categorized according to the ECW/ICW ratio quartiles.

### 2.4. Statistical Analyses

Data were analyzed using JMP pro (version 16.0; SAS Institute, Inc., Cary, NC, USA). The measured values were expressed as mean ± standard deviation or median (interquartile range). Statistical significance was assessed using a linear regression model for the continuous variables and Pearson’s chi-square test for the categorical variables. Correlations between the variables were determined using the Pearson product-moment correlation coefficient. Linear regression analysis was used to identify associations between the ECW/ICW ratio and natriuretic peptides and the left ventricular mass index. The explanatory variables for multivariate analysis were based on Japanese Society for Dialysis Research guidelines [20]: factors that increased the risk of developing new heart failure in patients undergoing dialysis included age, diabetes, history of coronary artery disease, decreased left ventricular systolic function, increased diastolic BP, hypoalbuminemia, and decreased Hb concentration. Alternatively, variables that had a significant correlation with these cardiac markers were analyzed to evaluate independent associations. Receiver operating characteristic (ROC) curve analysis was used to identify the best prognostic value of the ECW-to-ICW ratio for NT-proBNP (≥4000 or ≥8000 pg/mL) [21] and hANP (≥75 or ≥100 pg/mL). These were equivalent to the NT-proBNP cutoff values based on the regression equation between NT-proBNP and hANP [log_10_-transformed hANP = 0.3065 + 0.4283 × log_10_-transformed NT-proBNP]. *p* < 0.05 was considered statistically significant.

## 3. Results

### 3.1. Population Characteristics

The population characteristics of the patients (261 men and 107 women; mean age, 65 ± 12 years) are presented by the ECW/ICW ratio quartiles in Table 1. The quartile values for men and women were 0.611, 0.638, and 0.663, and 0.628, 0.650, and 0.674, respectively. Patients in the higher ECW/ICW ratio quartiles tended to be older and had a longer dialysis vintage, lower BMI, ultrafiltration volume, pre-dialysis diastolic BP, pulse rate, serum albumin, blood urea nitrogen, serum creatinine, serum phosphorus, total cholesterol, serum triglyceride, serum uric acid, hemoglobin levels, and GNRI, and higher post-dialysis systolic BP, CTR, serum chloride, C-reactive protein, NT-proBNP, and hANP levels in both men and women (*p* < 0.05).

As shown in Supplementary Tables S1 and S2, both men and women in the higher ECW/ICW ratio quartiles tended to have lower body weight, body surface area, TBW, ICW, ICW per BSA, and muscle mass, and had higher ECW per BSA. As shown in Supplementary Figure S1, both men and women in the higher ECW/ICW ratio quartiles tended to have a higher percentage of ECW content in TBW content at both times of pre- and post-hemodialysis. This trend was similar even if the ECW content in normal hydrated adipose tissue was excluded.

### 3.2. Association between Body Fluid Imbalance and Natriuretic Peptides

**Figure 1:**
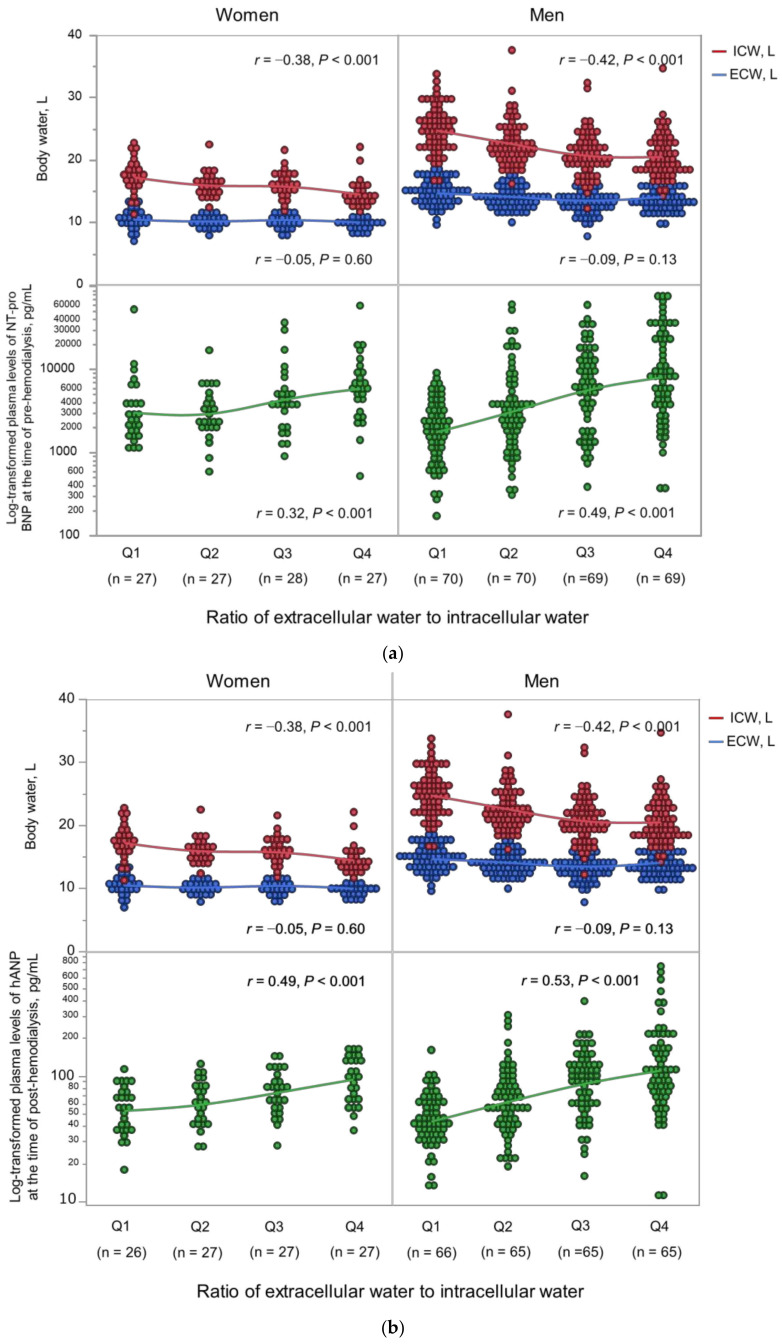
(**a**) Body fluid composition and NT-proBNP by the ECW/ICW ratio quartiles. (**b**) Body fluid composition and hANP by the ECW/ICW ratio quartiles. ECW, extracellular water; ICW, intracellular water; NT-proBNP, pre-dialysis *N*-terminal pro-B-type natriuretic peptide; hANP, post-dialysis human atrial natriuretic peptide.

**Figure 2:**
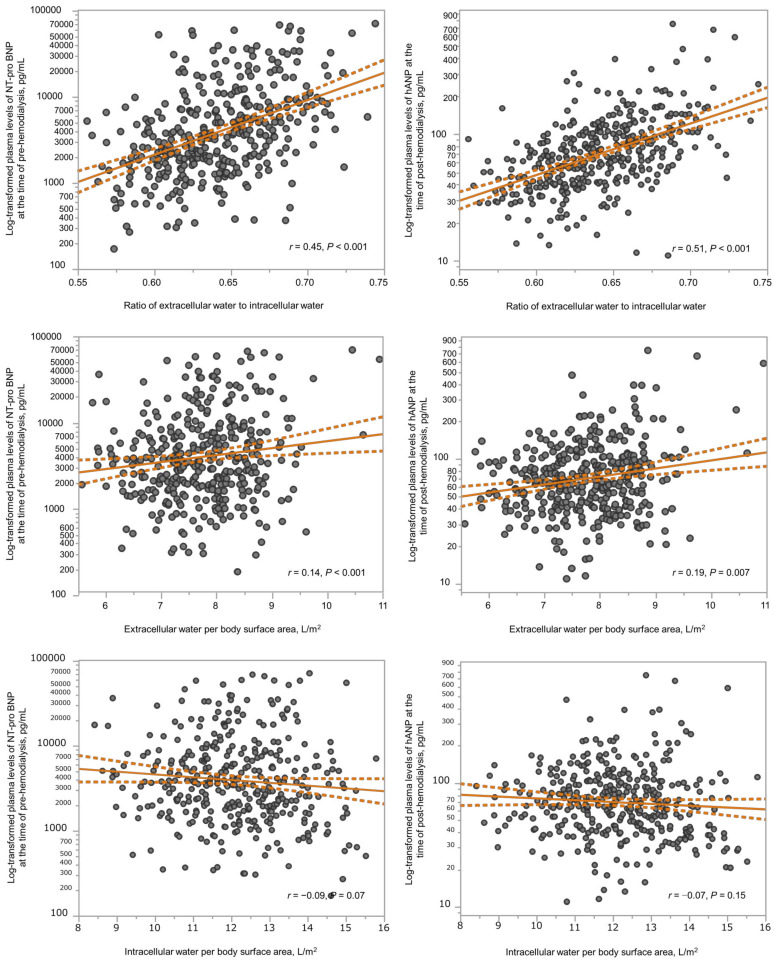
Scatter plots showing the correlations between the ECW/ICW ratio (upper X axes), the ECW per BSA (middle X axes), or the ICW per BSA (below X axes) and the log_10_-transformed natriuretic peptides (y axes). Solid lines and dashed lines show regression line and confidence interval, respectively. ECW, extracellular water; ICW, intracellular water; NT-proBNP, pre-dialysis *N*-terminal pro-B-type natriuretic peptide; hANP, post-dialysis human atrial natriuretic peptide.

Body fluid composition and log_10_-transformed natriuretic peptide levels according to the ECW/ICW ratio quartiles are shown in Figure 1. The ECW/ICW ratio had a significant negative correlation with the ICW content (men: *r* = −0.42 in men, *p* < 0.001; women: *r* = −0.38, *p* < 0.001), but not with the ECW content (men: *r* = −0.05, *p* = 0.60; *r* = −0.09, *p* = 0.13). Thus, the ECW/ICW ratio was primarily derived from decreased intracellular volume and not excess extracellular volume. The ECW/ICW ratio had significant positive correlations with the log_10_-transformed NT-proBNP (men: *r* = 0.49, *p* < 0.001; women: *r* = 0.32, *p* < 0.001) and log_10_-transformed hANP (men: *r* = 0.53, *p* < 0.001; women: *r* = 0.49, *p* < 0.001). The correlations between the ECW/ICW ratio, ECW per BSA, ICW per BSA, and log_10_-transformed natriuretic peptides are shown in Figure 2. In comparison to the ECW per BSA or ICW per BSA, the ECW/ICW ratio had a stronger correlation with the log_10_-transformed natriuretic peptides.

**Figure 3:**
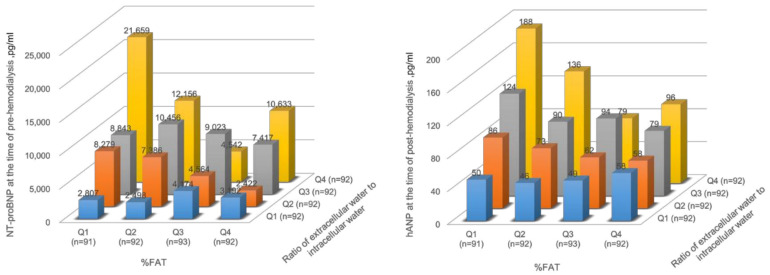
Association between body fluid imbalance and natriuretic peptides according to the fat percentage quartiles. NT-proBNP, pre-dialysis *N*-terminal pro-B-type natriuretic peptide; hANP, post-dialysis human atrial natriuretic peptide; Q1, Quatile1; Q2, Quatile2; Q3, Quatile3; Q4, Quatile4.

As shown in Figure 3, patients with a higher ECW/ICW ratio in the fat percentage quartiles 1 and 2 tended to have higher natriuretic peptide levels. However, the trend was lessened in the fat percentage quartile 3. In addition, the highest fat percentage quartile with a higher ECW/ICW ratio, which indicates sarcopenic obesity, also tended to have higher natriuretic peptide levels. The quartile values of fat percentage for men and women were 18.3, 24.2, and 29.5, and 21.8, 29.9, and 38.5, respectively.

### 3.3. Association between Body Fluid Imbalance and Echocardiographic Findings

Echocardiography findings performed within one year according to the ECW/ICW ratio quartiles are shown in Table 2. Patients in the higher ECW/ICW ratio quartile tended to have a wider LAD, narrower LVDd, thicker PWT and IVST, and heavier LVMI (*p* < 0.05).

### 3.4. Body Fluid Imbalance Is an Independent Associated Factor for Natriuretic Peptides and LVMI

In the linear regression analysis, the ECW/ICW ratio remained an independent associated factor for log_10_-transformed natriuretic peptides and LVMI (Table 3). We constructed receiver-operating characteristic curves to derive the cut-off values of the ECW/ICW ratio for these cardiac biomarkers. The best cutoff values of the ECW/ICW ratio for NT-pro BNP ≥ 4000 and ≥8000 pg/mL were 0.638 and 0.637 in men and 0.652 and 0.660 in women, respectively. The best cutoff values of the ECW/ICW ratio for hANP ≥ 75 and ≥100 pg/mL were 0.624 and 0.632 for men and 0.653 and 0.685 for women, respectively.

## 4. Discussion

This study revealed that the ICW-ECW fluid imbalance in patients undergoing hemodialysis was significantly associated with natriuretic peptides released from the heart in response to pressure and fluid volume and LVMI, an indicator of concentric left ventricular remodeling, despite the ECW/ICW ratio being primarily driven by decreased intracellular volume rather than by excess extracellular volume. That is, volume overload in patients undergoing hemodialysis may be characterized by a relative increase in ECW content with a decrease in ICW content by aging or muscle attenuation as well as an absolute increase in ECW content by sodium retention.

Obesity and metabolic syndromes affect the onset and progression of chronic kidney disease (CKD). Nutrition-related health problems in advanced CKD cause malnutrition in association with sodium retention, arterial stiffness, and inflammation. MIA syndrome is a serious clinical syndrome occurring in patients with advanced CKD [22]. Furthermore, malnourished patients on dialysis are prone to fluid accumulation.

In the intermittent hemodialysis regimen, fluid accumulates between dialysis sessions and is removed during dialysis sessions, just like the ebb and flow cycle of tides. Clinicians primarily set the fluid surface of the ebb tide after dialysis. The flow (IDWG) is determined primarily by oral sodium and water intake, residual urine output, and insensible fluid losses; it is often used as a marker of better nutrition or nonadherence to sodium and water restrictions [23]. Approximately 25–50% of patients undergoing dialysis do not achieve an adequate fluid volume status even after dialysis, leaving the patient in persistent chronic fluid overload status [2,23]. Hecking et al. emphasized that chronic fluid overload is more strongly associated with mortality risk than IDWG [24]. However, we often experience patients who have clinical signs of fluid overload but cannot lower their dry weight. In spite of patients in the higher ECW/ICW ratio quartiles having several clinical signs of fluid overload, such as higher post-dialysis systolic BP and larger CTR, clinical physicians seem to consider their post-dialysis weight as clinical dry weight. Nevertheless, natriuretic peptide levels in the higher ECW/ICW ratio quartiles tend to be higher.

The total fluid volume depends on age, sex, and body size. The ICW content may be associated with the muscle component in healthy individuals. In adulthood, the TBW content is relatively constant until the age of 40 years; thereafter, it gradually declines at the rate of 0.1–0.3 kg/yr until 70–80 years of age. The decreasing ICW slope with age is steeper than the decreasing ECW slope. In particular, there is a dramatic change in the balance between the ICW and ECW content after the age of 70 [13]. Approximately 75% of the muscles and viscera are composed of water [25]; organ aging [26] and sarcopenia [27,28] are associated with ICW loss. This universal cell volume loss expands the interstitial area, which leads to a mismatch in ICW-ECW content balance. We believe that this apoptotic fluid volume imbalance is less likely to preserve excess fluid because the cell area is replaced by interstitium in lean tissue; just as a small bottle already filled with water cannot store more water. In addition, this replaced fluid filling the interstitium probably cannot be removed by ultrafiltration. Indeed, in this present study patients with the higher ECW/ICW ratio quartiles tended to have a higher percentage of ECW content in TBW content at both times of pre- and post-hemodialysis. In our daily clinical practice, we think that a two-pronged approach is very important for normalizing the ECW/ICW ratio. First, we should try to remove excess ECW in patients who have clinical signs of fluid overload. Next, we should supply nutritional support to gain muscle mass for patients with elevated natriuretic peptides and higher ECW/ICW ratio along with a downward resetting of dry weight. A previous study has reported that the ECW/ICW ratio provides a prognostic value superior to that of B-type natriuretic peptide for the incidence of heart failure-related re-hospitalization and all events in patients hospitalized due to acute heart failure [29]. This study suggests that correcting excess ECW alone is not enough to prevent future heart failure. On the other hand, optimal dry weight setting in obese patients is difficult in a different way. ECW content in obese patients is increased even in the absence of fluid accumulation because 15–20% of adipose tissue is composed of extracellular fluid area. Furthermore, we speculate that adipose tissue may work as a buffer for fluid accumulation because adipose tissue expands the extracellular fluid area. For example, we often experience obese patients who have no symptoms even in fluid accumulation of 6.0–7.0 L. In addition, obese patients undergoing hemodialysis often have myocardial damage, which may affect natriuretic peptide levels. In the present study, the positive relationship between the ECW/ICW ratio and natriuretic peptides was uncertain in the higher fat percentage quartile. As a result, patients with the ICW-ECW fluid imbalance in the lower fat percentage were likely to have a noticeable clinical significance.

Natriuretic peptides are neurohormones synthesized in cardiac myocytes in response to increased left ventricular wall stress and stretching, which are reportedly associated with fluid volume [30,31]. However, several cohorts have reported that natriuretic peptides are inversely associated with BMI [32,33]. In addition, natriuretic peptide accumulation is associated with poor nutritional status and reduced survival among patients undergoing hemodialysis [16,34,35]. Lower natriuretic peptide levels might result from: (1) increased receptors in the adipose tissue or (2) suppression of either the synthesis or release of natriuretic peptides by a substance produced in the lean mass. Natriuretic peptides are correlated with high-sensitive C-reactive protein (hsCRP) and Interleukin-6 (IL-6) levels; hsCRP predicts the future loss of lean mass. In the present study, the ECW/ICW ratio was associated with the natriuretic peptide levels, LAD, and LVMI. These results suggest that the ICW-ECW fluid imbalance in patients undergoing hemodialysis may have an adverse effect on the reserve capacity for fluid accumulation. Patients undergoing hemodialysis with a low BMI reportedly have a higher prevalence of hypertension, poorer control of BP, and greater left ventricular hypertrophy [36].

The present study has several limitations. First, it was a retrospective analysis of 368 of 1094 patients (33.6%) in a four-center study in maintenance hemodialysis clinics; hence, generalization to the general population undergoing evaluation may not be valid. Second, echocardiographic findings were obtained from annual transthoracic echocardiographic examinations instead of the time of recruitment or the BIA measurements. Third, the quantitative assessment of overhydration and malnutrition using the ECW/ICW ratio was difficult. Fourth, natriuretic peptide levels are influenced by various factors, such as age and BMI. These confounding factors might have affected the association between the ECW/ICW ratio and natriuretic peptide levels; therefore, future studies are required to clarify these issues.

## 5. Conclusions

The fluid volume imbalance between ICW and ECW in patients undergoing hemodialysis was significantly associated with natriuretic peptide levels. Additionally, such patients may have different reserve capacities for fluid accumulation. We recommend that both sides of retention of cell volume and correction of the increase in ECW content might be important for normalizing fluid volume balance. Recognizing these changes in body composition due to aging and sarcopenia can then aid clinical decision-making for determining dry weight.

## Figures and Tables

**Table 1:** Population characteristics according to the post-hemodialysis ECW/ICW ratio quartiles.

Patients Characteristics	Post-Dialysis ECW/ICW Ratio				*p*
	Quartile 1 Men < 0.611 (*n* = 66) Women < 0.627 (*n* = 26)	Quartile 2 Men 0.611–0.637 (*n* = 65) Women 0.628–0.649 (*n* = 27)	Quartile 3 Men 0.638–0.662 (*n* = 65) Women 0.650–0.673 (*n* = 27)	Quartile 4 Men 0.663 ≦ (*n* = 65) Women 0.674 ≦ (*n* = 27)	
Age, years	56 (47–66)	66 (56–72)	71 (64–77)	73 (66–80)	<0.001
Diabetes mellitus, *n* (%)	36 (39)	40 (43)	46 (50)	46 (50)	0.37
Dialysis vintage, months	58 (4–409)	67 (8–446)	83 (7–537)	73 (6–549)	0.001
Cardiovascular disease, *n* (%)	18 (20)	9 (10)	22 (24)	21 (23)	0.06
Body mass index, kg/m^2^	24 (22–28)	22 (20–25)	21 (19–25)	21 (19–23)	<0.001
Ultrafiltration volume. L	3.6 (2.8–4.4)	2.7 (2.2–3.6)	3.0 (2.3–3.6)	2.6 (2.1–3.2)	<0.001
Ultrafiltration volume, % of body weight	5.5 (4.6–6.1)	4.9 (3.9–5.8)	5.3 (4.1–6.2)	4.8 (3.8–5.8)	0.044
Pre-dialysis systolic BP, mmHg	142 (128–160)	148 (131–159)	148 (132–165)	141 (125–160)	0.38
Post-dialysis systolic BP, mmHg	128 (118–138)	142 (120–155)	142 (124–163)	150 (130–165)	<0.001
Pre-dialysis diastolic BP, mmHg	82 (71–91)	78 (68–84)	76 (68–84)	72 (65–80)	<0.001
Post-dialysis diastolic BP, mmHg	75 (70–86)	78 (68–88)	76 (66–85)	75 (65–85)	0.31
Pulse rate, /min	72 (64–81)	70 (64–78)	70 (62–77)	68 (60–74)	<0.001
Serum albumin, mg/dL	3.7 (3.5–3.9)	3.6 (3.5–3.8)	3.5 (3.4–3.7)	3.5 (3.2–3.7)	<0.001
Blood urea nitrogen, mg/dL	65 (54–73)	58 (47–69)	57 (48–68)	55 (45–62)	<0.001
Serum creatinine, mg/dL	12.14 (10.48–13.85)	10.87 (9.79–12.06)	9.51 (8.74–10.94)	8.70 (7.54–971)	<0.001
Serum sodium, mEq/L	139 (137–141)	139 (137–140)	139 (137–141)	139 (138–141)	0.36
Serum potassium, mEq/L	4.8 (4.3–5.5)	4.9 (4.4–5.4)	4.4 (4.4–5.2)	4.3 (4.3–5.2)	0.25
Serum chloride, mEq/L	103 (100–104)	103 (102–105)	104 (102–106)	104 (102–106)	<0.001
Serum calcium, mg/dL	8.7 (8.4–8.9)	8.7 (8.3–8.9)	8.6 (8.2–8.9)	8.5 (8.1–8.9)	0.06
Serum phosphorus, mg/dL	6.0 (5.2–6.9)	5.4 (4.8–6.1)	5.4 (5.0–6.1)	5.4 (4.6–6.1)	<0.001
Total cholesterol, mg/dL	170 (153–195)	167 (143–196)	163 (142–196)	149 (129–175)	<0.001
Triglyceride, mg/dL	139 (92–218)	107 (69–151)	99 (63–140)	82 (63–104)	<0.001
Blood glucose, mg/dL	109 (91–154)	118 (96–159)	116 (98–151)	112 (99–144)	0.63
Uric acid, mg/dL	8.5 (7.5–9.6)	7.9 (7.3–85)	7.4 (6.7–8.2)	7.2 (6.4–8.1)	<0.001
Hemoglobin, g/dL	11.4 (10.9–12.1)	11.1 (10.7–11.9)	11.2 (10.6–11.7)	10.9 (10.3–11.4)	<0.001
C-reactive protein, mg/dL	0.13 (0.04–026)	0.09 (0.04–0.27)	0.09 (0.05–0.22)	0.16 (0.06–0.40)	0.046
Intact PTH, pg/mL	172 (111–226)	142 (92–226)	147 (88–210)	164 (104–204)	0.72
β_2_MG, mg/L	27 (24–29)	27 (24–30)	26 (23–32)	26 (23–32)	0.25
Kt/Vurea	1.73 (1.56–1.97)	1.87 (1.67–2.13)	1.85 (1.70–2.09)	1.80 (1.68–2.05)	0.06
Geriatric nutritional risk index	106 (95–109)	96 (91–104)	94 (89–100)	92 (87–98)	<0.001
NT-proBNP, pg/mL	1995 (1210–3678)	2810 (1795–5338)	5010 (2080–11,000)	6670 (3453–17,525)	<0.001
hANP, pg/mL	43 (34–66)	58 (42–84)	88 (59–118)	103 (67–153)	<0.001
CTR in men (*n* = 265), %	49.3 ± 4.7	49.3 ± 5.6	50.7 ± 4.2	51.6 ± 4.9	0.006
CTR in women (*n* = 107), %	51.0 ± 4.0	52.2 ± 3.2	52.6 ± 4.5	54.6 ± 4.5	<0.001

ECW/ICW, the extracellular to intracellular water; BP, pre- and post-dialysis blood pressure; PTH, intact parathyroid hormone; β_2_MG, β_2_-microglobulin; Kt/V, single pool Kt/Vurea; NT-proBNP, pre-dialysis *N*-terminal pro-B-type natriuretic peptide; hANP, post-dialysis human atrial natriuretic peptide; CTR, cardio-thoracic index.

**Table 2:** Echocardiographic findings according to the post-dialysis extracellular to intracellular water ratio quartiles.

Echocardiographic Findings	Post-Dialysis Extracellular Water to Intracellular Water Ratio				*p*
	Quartile 1 Men < 0.611 (*n* = 66) Women < 0.628 (*n* = 27)	Quartile 2 Men 0.611–0.637 (*n* = 67) Women 0.628–0.649 (*n* = 26)	Quartile 3 Men 0.638–0.662 (*n* = 66) Women 0.650–0.673 (*n* = 27)	Quartile 4 Men 0.663 ≦ (*n* = 66) Women 0.674 ≦ (*n* = 27)	
LAD, mm	36 (33 to 38)	37 (34 to 42)	38 (34 to 42)	38 (35 to 43)	0.002
LVDd, mm	46.4 ± 7.3	45.2 ± 6.4	45.8 ± 7.1	45.2 ± 6.5	0.036
LVDs, mm	30.2 ± 5.8	29.5 ± 5.5	30.1± 6.1	30.0 ± 6.8	0.31
PWT, mm	10.5 (9.3 to 11.7)	10.5 (9.1 to 12.0)	11.0 (10.0 to 12.0)	11.2 (9.6 to 12.8)	0.005
IVST, mm	10.7 ± 2.2	11.0 ± 2.1	11.1 ± 2.0	11.5 ± 2.2	0.002
EF	64 (59 to 70)	64 (60 to 70)	63 (56 to 70)	64 (59 to 69)	0.58
LVMI, g/m^2^	99 (79 to 127)	102 (79 to 128)	110 (95 to 132)	115 (96 to 137)	<0.001

LAD, left atrial dimension; LVDd, left ventricular end-diastolic diameter; LVDs, left ventricular end-systolic diameter; PWT, left ventricular posterior wall thickness; IVST, interventricular septum thickness; EF, ejection fraction; LVMI, left ventricular mass index.

**Table 3:** Extracellular to intracellular water ratio as an independent factor associated with NT-proBNP, hANP, and LVMI.

Variables	Unstandardized B (95% CI)	Standardized β	*p*
***Pre-dialysis Log_10_NT-proBNP***			
Unadjusted	6.34 (5.05, 7.63)	0.45	<0.001
Age and gender-adjusted	6.15 (4.66, 7.63)	0.44	<0.001
Multivariable-adjusted ^1^	4.66 (2.90, 6.42)	0.34	<0.001
***Post-dialysis Log_10_hANP***			
Unadjusted	4.08 (3.38, 4.78)	0.51	<0.001
Age and gender-adjusted	3.98 (3.17, 4.78)	0.50	<0.001
Multivariable-adjusted ^2^	3.15 (2.19, 4.10)	0.40	<0.001
***LVMI in*** **echocardiography**			
Unadjusted	163 (69, 308)	0.17	<0.001
Age and gender-adjusted	199 (91, 299)	0.21	<0.001
Multivariable-adjusted ^3^	183 (70, 295)	0.20	0.002

^1^ Age, dialysis vintage, diabetes, history of cardiovascular disease, body mass index, percentage of ultrafiltration volume, diastolic BP, serum albumin, triglyceride, total cholesterol, uric acid, BUN, creatinine, β2MG, CRP, Hb, and EF were entered into the multivariable model. ^2^ Age, DM, history of cardiovascular disease, body mass index, percentage of ultrafiltration volume, diastolic BP, serum albumin, triglyceride, total cholesterol, uric acid, BUN, creatinine, CRP, Hb, and EF were entered into the multivariate model. ^3^ Age, gender, DM, history of cardiovascular disease, percentage of ultrafiltration volume, systolic BP, serum albumin level, Hb level, and EF were entered into the multivariable model. NT-proBNP, pre-dialysis *N*-terminal pro-B-type natriuretic peptide; hANP, post-dialysis human atrial natriuretic peptide; CTR, the cardio-thoracic index; LVMI, left ventricular mass index; BP, blood pressure; BUN, blood urea nitrogen; β2MG, β2-microglobulin; CRP, C-reactive protein; Hb, Hemoglobin; EF, ejection fraction; DM, diabetes mellitus.

## Data Availability

The datasets generated and analyzed during the current study are available from the corresponding author on reasonable request.

## Supplementary Materials

The following supporting information can be downloaded at: https://www.mdpi.com/article/10.3390/nu15051274/s1. Table S1: Body fluid composition by man quartiles of extracellular water to intracellular water ratio at the time of post-hemodialysis; Table S2: Body fluid composition by woman quartiles of extracellular water to intracellular water ratio at the time of hemodialysis; Figure S1: Percentage of extracellular water in total body water by quartiles of extracellular water to intracellular water ratio at both time of pre- and post-hemodialysis.
