# Supplementary material for: Association between Intra- and Extra-Cellular Water Ratio Imbalance and Natriuretic Peptides in Patients Undergoing Hemodialysis
Source: Nutrients. 2023 Mar 3;15(5):1274. doi: 10.3390/nu15051274 (PMC10005491; doi:10.3390/nu15051274)
Supplement: Supplementary file 1 [file nutrients-15-01274-s001.zip › nutrients-2227043-supplementary.pdf]

Supplemental Table S1. Body fluid composition according to the post-hemodialysis ECW/ICW ratio quartiles in men

Body fluid composition	Post-dialysis ECW/ICW ratio				
	Quartile 1<br>Men <0.611<br>(n=66)	Quartile 2<br>Men<br>0.611–0.637<br>(n=65)	Quartile 3<br>Men<br>0.658–0.662<br>(n=65)	Quartile 4<br>Men 0.663≦<br>(n=65)	*P for trend*
Body weight, kg	70.6<br>(63.3–79.2)	62.8<br>(58.2–69.8)	60.2<br>(58.2–69.8)	57.9<br>(52.3–67.8)	<0.001
Body surface area, m^2	1.81<br>(1.71–1.96)	1.72<br>(1.64–1.81)	1.68<br>(1.56–1.80)	1.64<br>(1.54–1.79)	<0.001
Total body water, l	39.3<br>(35.2–43.6)	35.8<br>(33.0–40.0)	33.5<br>(31.0–37.0)	33.5<br>(31.0–38.2)	0.007
Total body water, l per BSA	21.7<br>(20.3–22.8)	21.0<br>(19.7–21.9)	20.0<br>(18.8–21.3)	20.5<br>(19.3–21.8)	0.37
Intracellular water, l	24.7<br>(22.0–27.2)	22.0<br>(20.4–24.7)	20.3<br>(18.8–22.5)	19.8<br>(18.4–22.7)	<0.001
Intracellular water, l per BSA	13.5<br>(12.8–14.9)	12.9<br>(12.2–13.5)	12.1<br>(11.3–12.9)	12.1<br>(11.5–12.9)	0.002
Extracellular water, l	14.7<br>(13.2–16.4)	13.7<br>(12.7–15.3)	13.3<br>(12.2–14.6)	13.5<br>(12.6–15.5)	0.60
Extracellular water, l per BSA	8.0<br>(7.6–8.5)	8.0<br>(7.6–8.4)	7.9<br>(7.4–8.4)	8.4<br>(7.8–8.8)	0.009
Muscle, kg	10.7<br>(9.5–11.8)	9.5<br>(8.9–10.7)	8.8<br>(8.1–10.7)	8.6<br>(7.9–9.9)	<0.001
%Muscle, %	14.9<br>(14.0–16.1)	14.9<br>(13.9–16.0)	14.3<br>(13.6–15.6)	14.7<br>(13.7–16.1)	0.11
Mineral, kg	3.57<br>(3.24–3.95)	3.27<br>(2.94–3.66)	3.06<br>(2.78–3.36)	3.01<br>(2.74–3.35)	<0.001
%Mineral, %	5.0<br>(4.7–5.4)	5.1<br>(4.7–5.5)	4.9<br>(4.6–5.5)	5.2<br>(4.7–5.8)	0.46
Fat, kg	18.7<br>(12.6–23.3)	14.9<br>(10.9–20.3)	15.2<br>(11.0–21.2)	12.9<br>(8.8–19.1)	0.08
%Fat, %	25.6<br>(18.7–30.2)	24.1<br>(18.2–29.0)	26.7<br>(19.0–30.7)	22.9<br>(16.0–28.2)	0.32

ECW/ICW, the extracellular to intracellular water; BSA, body surface area

Supplemental Table S2. Body fluid composition according to the post-hemodialysis ECW/ICW ratio quartiles in men

Body fluid composition	Post-dialysis ECW/ICW ratio Quartile 1 Women <0.627 (n=26)	Quartile 2 Women 0.628–0.649 (n=27)	Quartile 3 Women 0.650–0.673 (n=27)	Quartile 4 Women 0.674≦ (n=27)	*P for trend*
Body weight, kg	52.8 (40.4 to 66.7)	49.3 (42.1 to 59.6)	48.4 (44.5 to 59.5)	46.8 (41.9 to 52.3)	0.006
Body surface area, m^2	1.53 (1.42 to 1.66)	1.45 (1.35 to 1.58)	1.47 (1.39 to 1.59)	1.41 (1.30 to 1.47)	0.002
Total body water, l	27.6 (25.4 to 30.9)	25.3 (23.8 to 27.3)	25.8 (24.2 to 28.4)	24.1 (22.1 to 25.9)	0.007
Total body water, l per BSA	18.2 (17.0 to 19.0)	18.0 (16.2 to 18.7)	17.4 (16.5 to 18.3)	17.3 (16.0 to 18.6)	0.37
Intracellular water, l	17.2 (16.0 to 19.1)	15.5 (13.9 to 16.7)	15.6 (14.6 to 18.2)	14.2 (12.9 to 15.4)	<0.001
Intracellular water, l per BSA	11.4 (10.6 to 11.8)	11.0 (10.4 to 11.4)	10.5 (9.9 to 11.1)	10.1 (9.5 to 11.1)	0.002
Extracellular water, l	10.5 (9.5 to 11.7)	9.8 (9.2 to 10.6)	10.2 (9.6 to 11.3)	9.8 (9.2 to 10.5)	0.60
Extracellular water, l per BSA	6.8 (6.5 to 7.2)	7.0 (6.6 to 7.3)	6.9 (6.5 to 7.3)	7.1 (6.5 to 7.6)	0.009
Muscle, kg	7.5 (7.0 to 8.2)	6.7 (6.3 to 7.2)	6.7 (6.3 to 7.4)	6.1 (5.7 to 6.6)	<0.001
%Muscle, %	13.3 (12.0 to 14.7)	13.1 (11.9 to 15.7)	13.6 (11.6 to 15.4)	13.6 (11.9 to 14.7)	0.84
Mineral, kg	2.54 (2.35 to 3.04)	2.45 (2.28 to 2.74)	2.46 (2.27 to 2.66)	2.37 (2.20 to 2.63)	0.34
%Mineral, %	4.8 (4.2 to 5.4)	4.9 (4.5 to 6.0)	5.0 (4.1 to 5.8)	5.3 (4.9 to 5.8)	0.020
Fat, kg	16.3 (13.0 to 26.8)	17.7 (8.4 to 20.8)	14.0 (9.2 to 24.0)	12.5 (10.2 to 19.3)	0.06
%Fat, %	32..6 (25.8 to 39.4)	32.0 (19.8 to 37.2)	28.9 (21.1 to 40.0)	28.4 (22.6 to 36.9)	0.30

ECW/ICW, the extracellular to intracellular water; BSA, body surface area

Supplemental Figure S1. ECW/TBW ratio according to the post-hemodialysis ECW/ICW ratio quartiles

ATM, adipose tissue mass; ECW, extracellular water; ICW, intracellular water; TBW, total body water; UF, ultrafiltration

Foot note, ECW in adipose tissue was calculated as adipose tissue contains about 15% ECW.

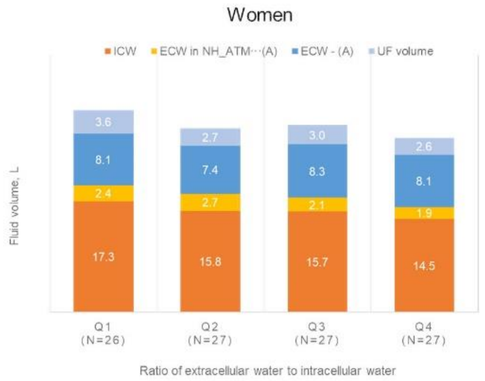

	Q1	Q2	Q3	Q4
Post-hemodialysis ECW - (A)/TBW ratio	0.290	0.287	0.318	0.332
Post-hemodialysis ECW/TBW ratio	0.378	0.390	0.398	0.408
(ECW - (A) + UF volume) /TBW ratio	0.403	0.391	0.419	0.425
(ECW + UF volume)/TBW ratio	0.449	0.448	0.460	0.464

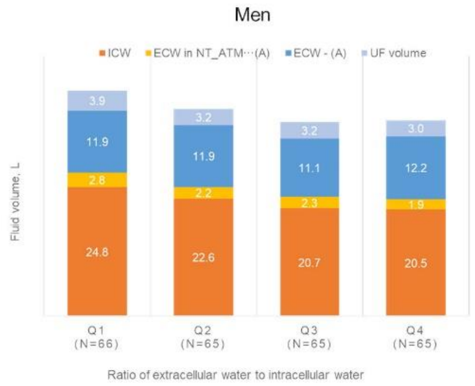

	Q1	Q2	Q3	Q4
Post-hemodialysis ECW - (A)/TBW ratio	0.301	0.323	0.326	0.352
Post-hemodialysis ECW/TBW ratio	0.372	0.384	0.393	0.408
(ECW - (A) + UF volume) /TBW ratio	0.389	0.400	0.409	0.425
(ECW + UF volume)/TBW ratio	0.429	0.434	0.445	0.455
